# The Effects of Dexmedetomidine on Secondary Acute Lung and Kidney Injuries in the Rat Model of Intra-Abdominal Sepsis

**DOI:** 10.1155/2013/292687

**Published:** 2013-02-13

**Authors:** Uğur Koca, Çimen Gülben Olguner, Bekir Uğur Ergür, Emel Altekin, Aydın Taşdöğen, Seden Duru, Pelin Girgin, Kerim Gündüz, Serap Cilaker Mıcılı, Seda Güzeldağ, Muhammed Akkuş

**Affiliations:** ^1^Department of Anaesthesiology and Reanimation, School of Medicine, Dokuz Eylül University, Inciralti, 35340 İzmir, Turkey; ^2^Department of Histology and Embryology, School of Medicine, Dokuz Eylül University, İzmir, Turkey; ^3^Department of Biochemistry and Clinical Biochemistry, School of Medicine, Dokuz Eylül University, İzmir, Turkey; ^4^Department of Anaesthesiology and Reanimation, Şifa Hospital, 35240 İzmir, Turkey; ^5^Department of Anaesthesiology and Reanimation, Samandağ State Hospital, 31800 Hatay, Turkey

## Abstract

In the present study, the effects of dexmedetomidine on secondary lung and kidney injuries were studied in the rat model of intra-abdominal sepsis by immunohistological and biochemical examinations. We measured serum creatinine, kidney tissue malondialdehide and plasma neutrophil gelatinase-associated lipocalin levels. In order to evaluate tissue injury we determined kidney tissue mononuclear cell infiltration score, alveolar macrophage count, histological kidney and lung injury scores and kidney and lung tissue immunoreactivity scores. We demonstrated that dexmedetomidine attenuates sepsis-induced lung and kidney injuries and apoptosis in the rat model of sepsis. There is still need for comparative studies in order to determine the effects of dexmedetomidine on organ functions in early human sepsis.

## 1. Introduction

Sepsis is a clinical syndrome characterized by a systemic inflammatory response triggered by an infection. The induction of a natural immune response leads to catastrophic effects in sepsis syndrome. This proinflammatory condition is called systemic inflammatory response syndrome (SIRS) [1]. Sepsis is characterized by activation of inflammatory, coagulation, and fibrinolytic cascades [2].

Sepsis shows a biphasic immunological pattern: the early hyperinflammatory phase is counterbalanced by an anti-inflammatory response which may lead to a hypoinflammatory state. The latter is associated with immunodeficiency that is characterised by monocytic deactivation [3].

Macrofages are the alternative source of injury. They can produce toxic oxygen metabolites, proteases, arachidonic acid metabolites, platelet-activating factor (PAF), and inflammation-regulating cytokines [4].

Apoptosis is a type of programmed cell death in which deoxyribonucleic acid (DNA) disintegration and cell death occur as a result of activation of death-inducing receptors or intracellular specific serine proteases (caspases) [1, 5–7]. Apoptosis might be the key manager in pro- and anti-inflammatory processes [1, 8, 9].

The role of apoptosis in the development of multiple organ dysfunction syndrome (MODS) and compensatory anti-inflammatory response syndrome (CARS) is not well established [1, 8, 9]. While apoptosis might be an important cause of immunologic suppression in sepsis, it can also have a benefit on survival by reducing the inflammatory response accompanying sepsis [8]. The mechanism by which the reduction of apoptosis leads to improved survival is not yet fully understood.

Dexmedetomidine is a potent lipophilic alpha-2 adrenoceptor agonist with an imidazole structure [10]. Dexmedetomidine exhibited anti-inflammatory and antiapoptotic properties in previous studies [11–13]. In this study, we aimed to examine the effect of dexmedetomidine on secondary renal and pulmonary injuries in the early sepsis by immunohistological and biochemical examinations. 

## 2. Materials and Methods

This study was performed after the approval of Dokuz Eylül University, School of Medicine (DEUMF), Experimental Animal Studies Ethic Board, by using the utilities of Multidisciplinary Experimental Animal Laboratory.

In this study, 21 Wistar albino adult male rats of 250–300 g were used. Subjects, obtained from DEUMF Experimental Animal Laboratory, were fed with standard chow food and water and housed in wire cages at room temperature under a 12 h day-night cycle. Within the preceding 12 hours of surgery only water was given. During the experiment, the body temperature of the animals was kept stable. 

### 2.1. Experimental Groups

Three groups of animals were used in this study: the sham-operated group (sham, *n* = 7) which underwent a laparotomy; the sepsis group (sepsis, *n* = 7) which underwent cecal ligation and perforation (CLP); the dexmedetomidine-treated group (sepsis + dex, *n* = 7) which underwent CLP and received dexmedetomidine 50 *μ*g/kg (precedex 100 *μ*g/mL, Abbot, İstanbul, Turkey) in 3 mL/100 g body weight normal saline intraperitoneally immediately after surgical procedure.

### 2.2. Anesthetic Procedure

Anesthesia was achieved by giving 50 mg/kg ketamine (*Ketalar*, Pfizer Pharma GMBH, and Germany) and 10 mg/kg xylazine hydrochloride (Alfazyne, %2, Alfasan International, 3440 AB, Woerden, The Netherlands) to subjects, intraperitoneally [14]. During the study, the same dose of anesthesia was reapplied as required and recorded, in order to maintain the immobility of subjects.

### 2.3. The Constitution of Experimental Sepsis Model by Cecal Ligation and Perforation

After an overnight fast, the rats were anesthetized. During the study, all animals were allowed to breathe spontaneously. After skin shaving and preparation of the abdominal wall with 10% povidone-iodine solution, a 2 cm midline laparotomy was performed. The cecum was then exposed, ligated just distally to the ileocecal valve to avoid intestinal obstruction, punctured tree times with an 18-gauge needle at the antimesenteric surface, gently squeezed to extrude a small amount of feces from the perforation sites, and returned to the abdominal cavity [15]. The abdominal cavity was then closed in two layers, animals in sepsis and sham groups received normal saline intraperitoneally (3 mL/100 g body weight) immediately after surgical procedure to prevent dehydration, and only animals in sepsis + dex group received dexmedetomidine 50 *μ*g/kg (precedex 100 *μ*g/mL, Abbot) in 3 mL/100 g body weight normal saline intraperitoneally. Sham-operated animals underwent the same surgical procedure except that the cecum was neither ligated nor punctured.

### 2.4. Experimental Protocol

At the end of 6 h after sepsis induction, abdominal cavity was opened, kidneys were removed, and than a midline sternotomy was performed the rats were exsanguinated by needle aspiration of the right ventricle, and the lungs and trachea were removed en bloc under anesthesia. Plasma samples, lungs, and kidneys were immediately frozen in liquid nitrogen and then stored at −80°C for later determinations of serum creatinine, plasma neutrophil gelatinase-associated lipocalin (NGAL), kidney tissue malondialdehide (MDA) levels, and for later tissue histopathologic and immunohistochemical examinations (light microscopic evaluations of the kidney and lung tissue injuries and apoptosis).

### 2.5. Serum Creatinine Level

Serum creatinine was measured with a quantitative kit on a Architect 16000 analyzer (Abbott Laboratories, USA). The serum creatinine results were expressed as mg/dL. 

### 2.6. Kidney Tissue Malondialdehide Level

Tissue homogenates are prepared by mechanical disruption of a tissue samples using the TissueLyser (QIAGEN) 5 min at 30 Hz in volume of 0.1 M phosphate buffer, pH 7.5. Tissue homogenates were then centrifuged at 10000 g 4°C for 5 min. The upper clear supernatants were transferred to a 2 mL eppendorf cup. Protein levels of tissue samples were measured with a quantitative kit on a Architect 16000 analyzer (Abbott Laboratories, USA). 

Concentrations of MDA in samples were determined by a high-pressure liquid chromatography method as described by Hong et al. [16], using a C-18 reversed phase column 5 *μ*M (250 × 4.6 mm I.D) and a mobile phase of a KH_2_PO_4_ (0.01 M)—30% methanol with fluorescent detector. The MDA results were expressed as *μ*mol/gr protein.

### 2.7. Plasma NGAL

Plasma NGAL levels were quantified using a commercially available NGAL Elisa kit (Boster Biological Technology, China) according to the manufacturer instruction. The *plasma NGAL* results were expressed as picogram/mL.

### 2.8. Histomorphological Procedures

#### 2.8.1. Light Microscopic Tissue Preparation

The tissue samples were fixed in 10% formalin in phosphate buffer for 24–48 h processed by routine histological methods and embedded in paraffin blocks. Then paraffin blocks were placed in rotary microtome (RM 2255, Leica, Germany), and sections of 4-5 *μ*M thickness were obtained. After deparaffinization and rehydration, sections were stained with haematoxylin (01562E, Surgipath, Bretton, Peter Borough, Cambridgeshire,UK), eosin (01602E, Surgipath, Bretton, Peter Borough, Cambridgeshire, UK) (H and E), periodic acid Schiff (PAS), and masson's trichrome stain (2049 GBL, İstanbul, Turkey). The H&E stained sections were used to evaluate the general morphology, and PAS stain was used to evaluate the basal membrane of the tubules. With this technique, we could also evaluate the brush border of the proximal tubules. The masson's trichrome stain was used to evaluate the collagen content of the parenchyma. With this technique, increase in blue color in the parenchyma assesses the increase in connective tissue.

### 2.9. Histomorphological Assessment of Lung and Kidney Tissues

To evaluate kidney and lung tissue sections, the sections were stained with H&E and PAS stains. Digital images were obtained from sections using a camera (Olympus DP71, Olympus Optical Co. Ltd, Tokyo, Japan) connected to a light microscope (Olympus BX51, Olympus Optical Co. Ltd, Tokyo, Japan) at an original magnification of ×20. Three nonoverlapping lung and kidney sections and a minimum of 30 lung fields were examined per animal. Each lung and kidney were evaluated histologically by two histologists blind to the groups. 

A grading system was used to score for the alveolar and parenchymal general morphological changes (alveolar structure, inflammation, thickening of the alveolar septum, alveolar macrophage, neutrophile, increased capillary permeability, hemorrhage, edema, and congestion). The grading system was scored according to these findings, whether there is absence (score 0) or presence (score 1 for mild, score 2 for moderate, score 3 for marked, and score 4 for diffuse) in the alveolar tissue [17]. In addition, the amount of alveolar macrophages in the alveolar septum and lumen were visualized digitally and counted in a given area (macrophage count/0.016 mm^2^). 

Structural changes in the kidney tissue sections were evaluated and scored for proximal tubule damages (tubular atrophy, tubular brush border loss, vacuolization, tubular dilatation, and cast formation), mononuclear cell infiltration, erythrocyte extravasation, interstitial structural changes, renal corpuscle morphology, and necrotic and apoptotic cells by light microscopy. Tubulointerstitial damage in obtained cross-sectional images was scored semiquantitatively. The scoring system was made noticing to these findings: 0 = none, 1 = 1–25%, 2 = 26–50%, 3 = 51–75%, and 4 = 76–100% applied [18].

### 2.10. Immunohistochemical Assessment of M30 and Active Caspase-3 Expression

To detect DNA fragmentation in epithelial cells in lung tissue M30 (Cytokeratin-18) and to evaluate apoptosis in kidney tissue active caspase-3 immunohistochemistry was applied to the paraffin sections. After deparaffinization and rehydration, sections were treated with 10 mM citrate buffer (Cat number AP-9003-125 Labvision) for five minutes. Then sections were incubated in a solution of 3% H_2_O_2_ for 5 min to inhibit endogenous peroxidase activity. They were then incubated with blocking solution. After sections were incubated in a humid chamber, overnight at +4°C with rat-specific anti-M30 antibody 1 : 100 (SC-32329, Santa Cruz Biotechnology, USA) and anticaspase-3 antibody active form 1 : 100 (AB3623, Millipore, Temecula, CA). Sections were then incubated with biotinylated IgG and then with streptavidin for 30 min each prepared according to kit instructions (İnvitrogen-Plus Broad Spectrum 85-9043). Sections were finally stained with DAB and counter-stained with mayer's hematoxylin and analyzed using a light microscope (Olympus BX51, Olympus Optical Co. Ltd, Tokyo, Japan). 

### 2.11. Semiquantification of Immunostaining Data

A grading system was used to score the quantity of anti-M30 and anticaspase-3 positive staining in the sections [19]. The score was defined as follows: 1 = remarkably little positive staining, 2 = moderate positive staining and between grade 1 and grade 3, 3 = strong positive staining, that was evenly distributed across the whole image, and 0 = no immunoreactivity. Each section was graded by two histologists blind to the treatments, and the average was taken. 

### 2.12. Statistics

Statistical Package for Social Sciences 15 (SPSS 15.0, Chicago, IL, USA) programme was used for statistical analysis. For evaluating the data, descriptive statistical methods were used with Kruskal-Wallis test in order to analyze numeric values because of the insufficient number of the group population. When a statistical difference was obtained between groups, Mann-Whitney *U* test was used to confirm the difference between two groups. A statistically significant difference was accepted at a *P* value of <0.05. All values were expressed as ((median; max–min), mean ± standard deviation).

## 3. Results

**Figure 1:**
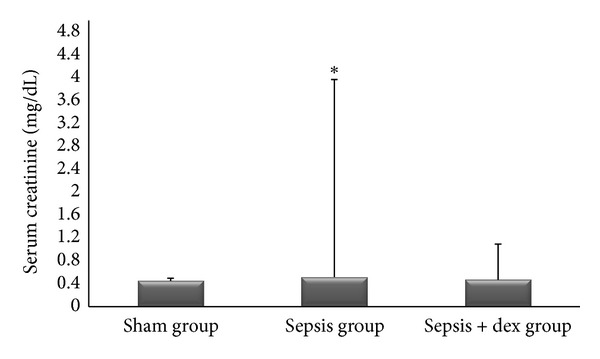
Serum creatinine levels in experimental groups. *P < 0.05 compared with sham and sepsis + dex groups.

**Figure 2:**
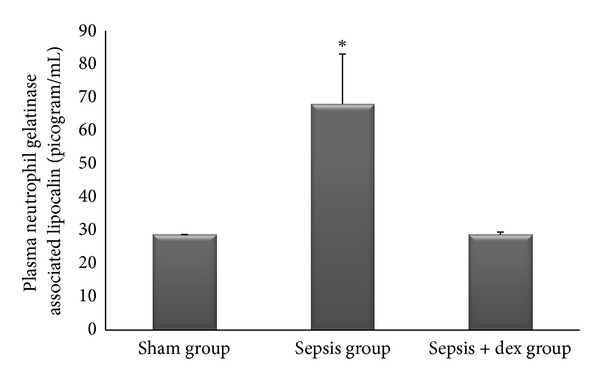
Plasma neutrophil gelatinase-associated lipocalin in experimental groups. *P < 0.05 compared with sham and sepsis + dex groups.

**Figure 3:**
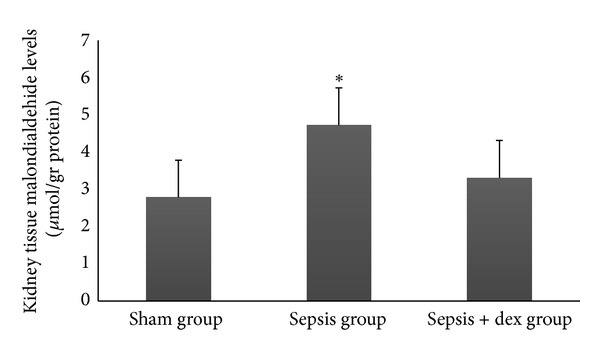
Kidney tissue malondialdehide levels in experimental groups. *P < 0.05 compared with sham and sepsis + dex groups.

**Figure 4:**
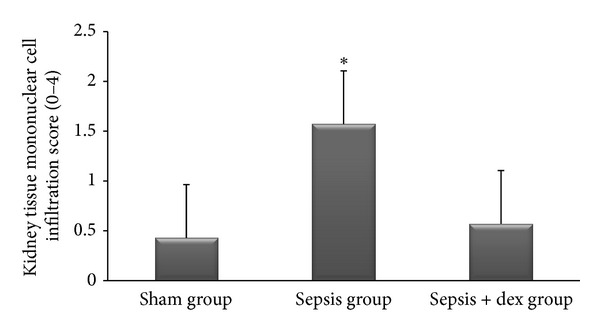
Kidney tissue mononuclear cell infiltration scores in experimental groups. *P < 0.05 compared with sham and sepsis + dex groups.

**Figure 5:**
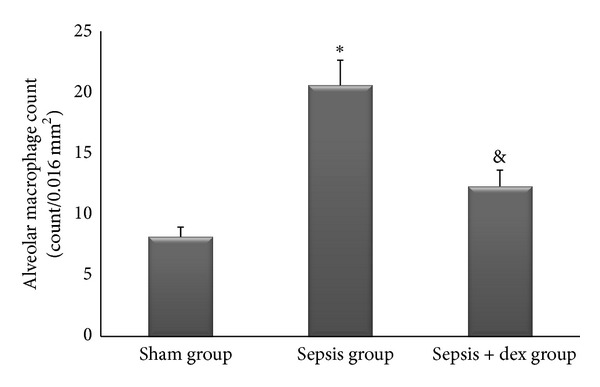
Alveolar macrophage counts in the experimental groups. *P < 0.05 compared with sham and sepsis + dex groups. ^&^ *P* < 0.05 compared with sham groups.

**Figure 6:**
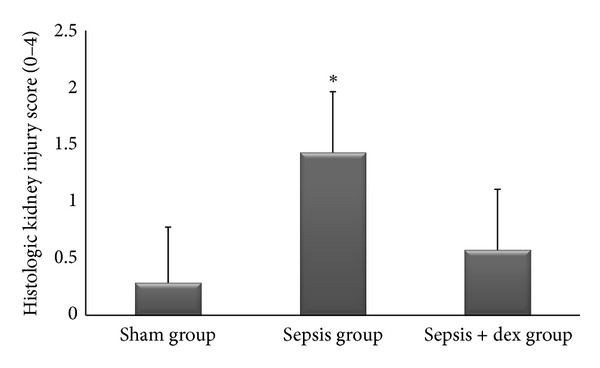
Histological kidney injury scores in experimental groups. *P < 0.05 compared with sham and sepsis + dex groups.

**Figure 7:**
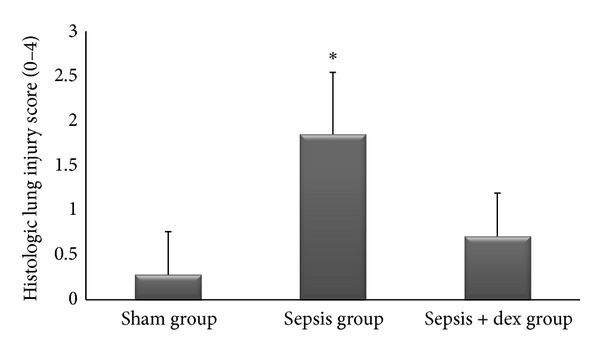
Histological lung injury scores in experimental groups. *P < 0.05 compared with sham and sepsis + dex groups.

**Figure 8:**
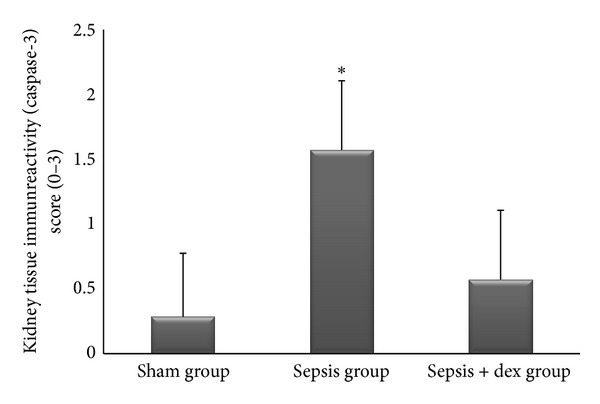
Kidney tissue immunoreactivity scores in experimental groups. *P < 0.05 compared with sham and sepsis + dex groups.

**Figure 9:**
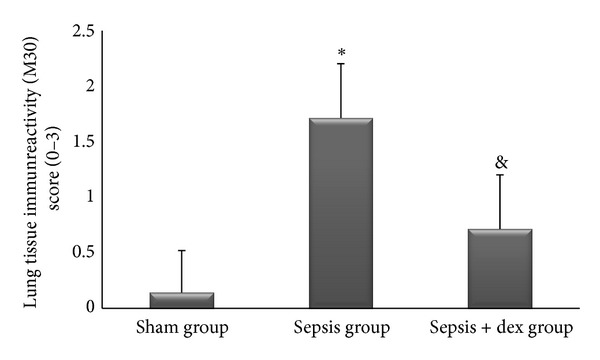
Lung tissue immunoreactivity scores in experimental groups. *P < 0.05 compared with sham and sepsis + dex groups. ^&^ *P* < 0.05 compared with sham group.

The ((median; max–min), mean ± standard deviation) of serum creatinine levels was ((0.44; 0.54–0.40), 0.45 ± 0.004), ((0.52; 0.54–0.48), 0.51 ± 3.44), and ((0.45; 0.52–0.42), 0.46 ± 0.62) mg/dL, of plasma NGAL levels was ((28.85; 33.35–24.15), 28.6 ± 0.02), ((69.80; 86.65–41.65), 67.9 ± 15.0), and ((27.50; 37.00–21.95), 28.7 ± 0.73) picogram/mL, and of kidney tissue malondialdehide levels were ((3.00; 3.50–1.50), and 2.78 ± 0.03), ((4.40; 5.80–3.90), 4.72 ± 5.17) and ((3.60; 4.00–2.50), 3.31 ± 0.68) *μ*mol/gr protein in the sham, sepsis, and sepsis + dex groups, respectively. Serum creatinine (Figure 1), plasma NGAL (Figure 2), and kidney tissue malondialdehide levels (Figure 3) in the sepsis group were significantly increased compared with the sham and sepsis + dex groups (*P* < 0.05). The change in the serum creatinine, plasma NGAL, and kidney tissue malondialdehide level in the sepsis + dex group was insignificant compared with the sham group (*P* = 0.38, *P* = 0.90, and *P* = 0.31, resp.). Figures 1–9 show mean ± standard deviation.

The ((median; max–min), mean ± standard deviation) of kidney tissue mononuclear cell infiltration scores was ((0; 1–0), 0.42 ± 0.53), ((2; 2–1), 1.57 ± 0.53), and ((1; 1–0), 0.57 ± 0.53), of alveolar macrophage counts were ((8.2; 8.8–7.6), 8.17 ± 0.78), ((21.2; 21.6–19.6), 20.57 ± 2.06), and ((12.2; 13–11.6), 12.34 ± 1.32), of histologic kidney injury scores was ((0; 1–0), 0.28 ± 0.48), ((1; 2–1), 1.42 ± 0.53), ((1; 1–0), 0.57 ± 0.53), of histologic lung injury scores was ((0; 1–0), 0.28 ± 0.48), ((2; 3–1), 1.85 ± 0.69), and ((1; 1–0), 0.71 ± 0.48), of kidney tissue immunreactivity (caspase 3) scores were ((0; 1–0), 0.28 ± 0.48), ((2; 2–1), 1.57 ± 0.53), ((1; 1–0), 0.57 ± 0.53), and of lung tissue immunreactivity (M30) scores was ((0; 1–0), 0.14 ± 0.37), ((2; 3–1), 1.71 ± 0.48), ((1; 2–0), 0.71 ± 0.48) in the sham, sepsis, and sepsis + dex groups, respectively. Kidney tissue mononuclear cell infiltration score (Figure 4), alveolar macrophage count (Figure 5), histologic kidney injury score (Figure 6), histologic lung injury score (Figure 7), kidney tissue immunreactivity (caspase 3) score (Figure 8), and lung tissue immunreactivity (M30) score (Figure 9) in the sepsis group were significantly increased compared with the sham and sepsis + dex groups (*P* < 0.05). Lung tissue immunreactivity (M30) score and alveolar macrophage count in the sepsis + dex group were significantly increased compared with the sham group (*P* < 0.05). The change in the kidney tissue mononuclear cell infiltration score, the histologic kidney and lung injury scores, and the kidney tissue immunreactivity (caspase 3) score in the sepsis + dex group were insignificant compared with the sham group (*P* = 0.71, *P* = 0.38, *P* = 0.21, and *P* = 0.38, resp.). 

**Figure 10:**
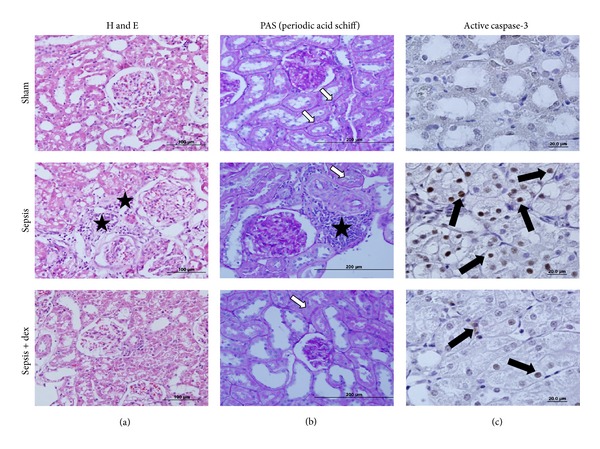
Representative histochemical and immunohistochemical staining in kidney tissue (H and E, PAS, and active caspase-3) micrographs of experimental groups. (a) represented H&E, (b) PAS, and (c) represented active caspase-3 immunohistochemistry stained sections. (★) showed mononuclear cell infiltration; (white arrows) resembled basal represented membrane regularity and brush border in proximal tubule endothelium; (black arrows) represented active caspase-3 positive cells.

In H&E staining of the kidney tissue sections, the sham group showed normal kidney histology. In the sepsis group, mononuclear cell infiltration around the glomerulus and capillaries, vasodilatation in veins, rare tubular degeneration, and cast formation in tubules were observed. (Figure 10(a)). In PAS staining, loss of brush border and irregularity in basal membrane were noticed in proximal tubular cells. Thickening of the parietal Bowman's capsule was also found. (Figure 10(b)). In sepsis + dex group, all these histomorphologic findings decreased when compared with sepsis group (Figures 10(a) and 10(b)). In active caspase-3 immunohistochemistry, caspase-3 immune positive cells were increased in sepsis group than in sham and sepsis + dex groups (Figure 10(c)).

**Figure 11:**
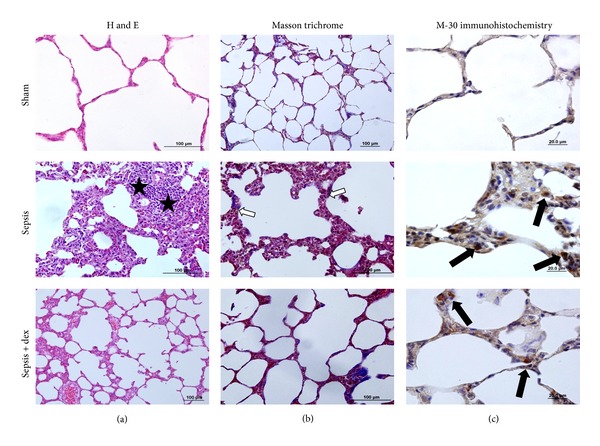
Representative histochemical and immunohistochemical staining in lung tissue (H and E, Masson's trichrome, and M30) micrographs of experimental groups. (a) represented H&E, (b) represented Masson's trichrome, and (c)  represented M30 immunohistochemistry stained sections. (★) showed mononuclear cell infiltration (white arrows) resembled collagen content of the parenchyma; (black arrows) represented M30 positive cells.

In H&E staining of the lung tissue sections, the sham group showed normal lung histology. In the sepsis group mononuclear cell infiltration, inflammation and thickening of the alveolar septum, increase in alveolar macrophage count, increased capillary permeability, hemorrhage, edema, and congestion were observed (Figure 11(a)). In masson's trichrome stain increase in the collagen content of the parenchyma of the lung tissue sections in sepsis group was found (Figure 11(b)). Besides, in M30 immunohistochemistry scoring, immunopositive epithelial cells were increased in sepsis group compared to sham and sepsis + dex groups (Figure 11(c)). 

## 4. Discussion

The present study demonstrated by biochemical and immunohistological examinations, that dexmedetomidine attenuates sepsis-induced lung and kidney injuries in the rat model of sepsis. 

Because CLP model mimics many features of clinical peritonitis, we used this model for sepsis induction [20]. Seely et al. [21] observed that renal blood flow decreased 61% by 6 hours after CLP and continuously flowing renal cortical capillaries decreased significantly from 69% to 48% by 6 hours with a 66% decrease in renal blood cell velocity and 57% decline in volumetric flow. Also, Wang et al. [22] demonstrated that CLP caused an increase in renal capillary permeability at 2 hours and a decrease in renal capillary perfusion at 4 hours in murine sepsis. Messaris et al. [23] observed that apoptotic renal tubular cell death was increased significantly 6 hours after CLP and declined subsequently. Apoptosis is a rapid process, taking approximately 4–6 hours from initiation to the structural disassembly of apoptotic cell [24]. Aunapuu et al. [25] demonstrated that histologically changes in kidneys starts in 2 hours after *Escherichia coli* injection. Based on these data, we aimed to investigate the tissue injuries at 6 hours after sepsis induction, and we harvested animals and removed tissues at 6 hours after CLP in the present study. 

Dexmedetomidine was used intraperitoneally at the dose of 25–100 *μ*g/kg in previous studies [26–28]. Also, we administrated dexmedetomidine at the dose of 50 *μ*g/kg intraperitoneally to rats in the present study. 

### 4.1. Serum Creatinine and NGAL Levels

In the present study, the serum creatinine level in sepsis group was significantly increased compared with the other groups. This result is consistent with the results of previous studies that also serum creatinine was measured at 6 hours after sepsis induction [29, 30]. Because of the hyperhrophy and hyperfiltration of noninjured nephrons and progressive increase of tubular creatinine secretion as kidney function deteriorates, the serum creatinine concentration could not increase until half of the kidney function is lost [31–33]. The serum creatinine is not an injury marker but rather a functional marker [34]. Regarding these data, we may consider that half of renal function was lost in sepsis group in the present study. 

LPS injection increases the plasma epinephrine and norepinephrine levels [35]. Norepinephrine profoundly constricts the afferent glomerular arterioles thus decreases the glomerular filtration pressure [36]. Cumming et al. [37] observed that the plasma norepinephrine level correlated inversely with glomerular filtration rate in nonhypotensive sepsis. They concluded that sympathetic nervous system could be involved in the renal response to sepsis. Activation of central postsynaptic alpha-2 adrenoceptors leads to inhibition of norepinephrine release [38]. So, we could conclude that, in the present study, the decrease in serum creatinine level in sepsis + dex group compared to sepsis group may be related to decrease in norepinephrine relaese in sepsis + dex group. We did not measure the serum norepinephrine level that limits the present study. The effect of dexmedetomidine on norepinephrine release was studied in previous studies. It has been shown that, alpha adrenoceptor subtypes involved in the regulation of catecholamine release from the adrenal medulla [39]. Taoda et al. [40] showed that dexmedetomidine reduced the release of norepinephrine by presynaptic alpha-2 agonist. Billings et al. suggested that dexmedetomidine preserves outer medullary blood flow by reducing the regional vascular resistance in radiocontrast-induced nephropathy in mice [41]. 

NGAL is a highly sensitive, specific, and predictive early biomarker for acute kidney injury [42, 43]. Bagshaw et al. [44] observed that septic acute kidney patients have higher plasma and urine NGAL levels compared with nonseptic acute kidney injury patients. However, Han et al. [45] observed that both serum and urinary NGAL levels increased in LPS-induced sepsis in rats. This result cosistent with our result. But, Han et al. [45] also found a correlation between urinary NGAL and NGAL mRNA in the injured kidney but not between plasma NGAL and NGAL mRNA. They concluded that urinary NGAL exactly reflects the change in renal NGAL expression, whereas plasma NGAL was not accurate in septic acute kidney injury. Also they concluded that plasma NGAL levels may be misleading in the diagnosis and monitoring of septic acute kidney injury. Paragas et al. [46] found that NGAL is present in the kidney, liver, spleen, lung, and trachea, which indicates that NGAL in the blood is not a good marker of septic acute kidney injury. Plasma NGAL is elevated in systemic response system, severe sepsis, and septic shock, and it should be used with caution as a marker of acute kidney injury in sepsis [47]. Regarding to these data, we may conclude that the elevation of serum NGAL level in the sepsis group compared to the other groups in the present study may be related to its release from all NGAL containing tissues (kidney, lung, spleen, liver, etc.) and not reflects only NGAL release from kidney. But, the increase in serum NGAL in sepsis group reflects the sepsis induced NGAL release from tissues in the present study. Serum NGAL measurement in our study limits the present study in terms of diagnosing septic acute kidney injury. We could not find any study that investigates the effects of dexmedetomidine on sepsis-induced acute kidney injury with NGAL analysis.

### 4.2. MDA Levels in the Kidney Tissue

The peroxidation of polyunsaturated fatty acids and the subsequent production of thiobarbituric acid reactive substances (TBARS) have been shown as common pathogenetic mechanisms involved in septic inflammatory processes where MDA, an end-product of fatty acid peroxidation, reacts with thiobarbituric acid [48]. During intra-abdominal sepsis models, a significant increase in MDA levels has been observed in kidney tissues [49, 50]. The generation of MDA is believed to originate in the inflammatory cells [48, 51]. Significantly elevated kidney tissue MDA level observed in the control group in the present study supports the supposition that the lipid peroxidation process occurs during sepsis-induced kidney injury. A decrease in kidney tissue MDA level in the sepsis + dex group suggests that dexmedetomidine attenuates lipid peroxidation. Dexmedetomidine reduced the brain and testis MDA levels in ischemia-perfusion models in previous studies [52–55]. However, we did not find any study that investigates the effects of dexmedetomidine on lipid peroxidation in sepsis.

### 4.3. Histomorphological Assessments of Lung and Kidney Tissues

Previous studies investigated the lung and kidney injuries using the haematoxylin-eosin staining and light microscope [56–59]. In these studies, significantly high histologic lung and kidney injury scores were observed in septic rats. Also, we found significantly increased histologic lung and kidney injury scores in the sepsis group compared with the other groups in the present study that consistent with the results of these previous studies. However, we did not find any studies that investigate the effects of dexmedetomidine on lung and kidney injuries by using haematoxylin-eosin staining and light microscope. In the present study, histologic lung and kidney injury scores in sepsis + dex group were significantly decreased compared to sepsis group. This results could be concluded as dexmedetomidine attenuates lung and kidney injury in sepsis. 

### 4.4. Kidney Tissue Mononuclear Cell Infiltration and Pulmonary Alveolar Macrophage Count

The mononuclear phagocyte system was involved in phagocytosis and participated many complex immunologic and inflammatory processes. Sepsis associates with increased production of cellular pro- and anti-inflammatory mediators by monocyte/macrophages. Alveolar macrophages play an essential role in the regulation of the pro- and anti-inflammatory events during sepsis induced acute lung injury [60–63]. 

In the present study, we found significantly increased alveolar macrophage count in the sepsis group compared with the other groups. There are many studies that investigated the anti-inflammatory properties of dexmedetomidine. Taniguchi et al. [12] showed that dexmedetomidine decreased plasma cytokine (TNF, IL6) concentration and infiltration of neutrophils in the airspace or in vessel walls of the lungs in sepsis. Shi et al. [64] showed the anti-inflammatory effects of dexmedetomidine in lung tissue in LPS-induced sepsis rat model. 

Dexmedetomidine modulates LPS induced TNF-*α* production on macrophages [65]. TNF plays an important role in septic acute kidney failure development [66, 67]. TNF receptor deficient mice are resistant to sepsis-induced acute renal failure development and exhibit less tubular apoptosis and mononuclear cell infiltration [67]. Apoptotic cells and bodies are recognized by specific surface receptors of tissue macrophages and digested [5]. Based on these data, we could conclude that the increased mononuclear cell infiltration in kidney tissue and in alveolar macrophage count in sepsis group compared with the other groups in the present study may be involved in sepsis induced tissue inflammation and high apoptotic activity. In the present study we did not measure cytokine levels, which limit the study. 

### 4.5. Apoptosis in the Lung and Kidney Tissues

Apoptosis is a type of programmed cell death in which DNA disintegration and cell death occur as a result of activation of death-inducing receptors or intracellular specific serine proteases (caspases) [1, 7]. 

The gold standard of the diagnosis of apoptosis is morphologic/ultrastructural evaluation [68]. The determination of apoptosis with hematoxylin/eosin staining and light microscopy is sensitive, specific, and cheapest way [68]. In hematoxylin/eosin stained sections apoptotic cells and bodies can be seen as dense nuclear fragments and a condensed eosinophilic (affinity for eosin, pink, and orange) cytoplasm [69]. 

Caspase cleaved cytokeratin 18 (CK18) is an early marker of apoptosis. It can be recognized in epithelial cells using a monoclonal antibody (M30) directed against this neo-epitope. CK18 is cleaved by several caspases (caspase-3, -6, -7, and -9) during apoptosis, and is dependent of single caspase activation [70, 71]. Since M30 neoepitope occurs in the early phase of the apoptotic cascade, it can be used in determination of induction or inhibition of apoptotic process [72, 73]. Based on these data, M30 antibody was used in the present study for the first time in order to evaluate the effects of dexmedetomidine on epithelial apoptosis in lung tissue during sepsis. 

A significant increase in M30 positive cells has been shown in lung tissue in CLP-induced sepsis compared to sham group, previously described [48, 74]. In the present study, the significant increase in pulmonary M30 positive cells in sepsis and in sepsis + dex groups compared to sham group indicates the presence of epithelial cell-specific expression of apoptosis in sepsis. Also, the significant decrease in pulmonary M30-positive cells in dex + sepsis group compared to the sepsis group indicates that dexmedetomidine reduces epithelial apoptosis in lung tissue in sepsis.

In the intrinsic pathway, death ligand binding (Fas L, TNF-*α*, ect.) results in the activation of initiator caspases (caspase-8 and -10). Initiator caspases subsequently activate the effector caspases (caspase-3, -6 and -7) [75]. Cytosolic caspase-3 activation is regulated by both TNF-*α* receptor mediated exrtinsic and intrinsic apoptotic cascades [76]. Apoptosis in renal cells occurs through TNF-*α* and Fas ligand-mediated pathways during sepsis [67, 77–80]. It has been shown that TNF-*α* receptor deficient mice are resistant to development of endotoxin-mediated acute renal failure and exhibit less tubular apoptosis and less mononuclear cell infiltration [67]. Messaris et al. [23] investigated the apoptotic death of renal tubular cells in rat CLP model of sepsis. They found that apoptotic renal tubular cell death increased significantly 6 hours after CLP and declined subsequently. They also observed that, cell initiating apoptosis were significantly more common, and caspase-8 protein immunodetection and Bax protein expression were increased at 6 hours after CLP. Additionally, in situ localization of cleaved caspase-3 has found some favor for histological labeling of cells in apoptosis [76]. Based on these data, we used caspase 3 immunohistochemical staining for determining the apoptosis in renal tissue. 

The present study investigated the effects of dexmedetomidine on renal cell apoptosis in sepsis by immunohistochemical assessment of active caspase-3 expression, for the first time. Qiao et al. [81] investigated the effects of dexmedetomidine and midazolam on splenic caspase 3 expression in sepsis. They showed that dexmedetomidine decreased splenic caspase 3 expression in CLP model of sepsis. Sanders et al. [13] showed that dexmedetomidine inhibited isoflurane-induced caspase-3 expression in cerebral cortex in rats. Engelhard et al. [82] demonstrated that dexmedetomidine reduced proapoptotic proteins and increased antiapoptotic proteins in cerebral ischemia reperfusion model in rats. Dexmedetomidine upregulates AKT/protein kinase B, and extracellular regulated signaling kinase and Bcl-2 [82–85]. In the present study, we found significantly increased caspase 3 positive staining proximal tubule epithelial cells in sepsis group compared to the other groups that indicate that CLP induced sepsis causes renal cell apoptosis and dexmedetomidine decreases it. 

In conclusion, dexmedetomidine seems to be a favorable sedative agent because of its anti-inflammatory and antiapoptotic properties. In our opinion, there is still need for comparative studies in order to determine the effects of dexmedetomidine on organ functions in early human sepsis.

## References

[1] Oberholzer C, Oberholzer A, Clare-Salzler M, Moldawer LL (2001). Apoptosis in sepsis: a new target for therapeutic exploration. *FASEB Journal*.

[2] Cohen J (2002). The immunupathology of sepsis. *Nature*.

[3] Kox WJ, Bone RC, Krausch D (1997). Interferon gamma-1b in the treatment of compensatory anti-inflammatory response syndrome: a new approach: proof of principle. *Archives of Internal Medicine*.

[4] Marriott HM, Dockrell DH (2007). The role of the macrophage in lung disease mediated by bacteria. *Experimental Lung Research*.

[5] Martin TR, Hagimato N, Nakamura M, Matute-Bello G (2005). Apoptosis and epithelial injury in the lungs. *Proceedings of the American Thoracic Society*.

[6] Hassoun HT, Kone BC, Mercer DW, Moody FG, Weisbrodt NW, Moore FA (2001). Post-injury multiple organ failure: the role of the gut. *Shock*.

[7] Mainous MR, Ertel W, Chaudry IH, Deitch EA (1995). The gut: a cytokine-generating organ in systemic inflammation?. *Shock*.

[8] Hotchkiss RS, Swanson PE, Cobb JP, Jacobson A, Buchman TG, Karl IE (1997). Apoptosis in lymphoid and parenchymal cells during sepsis: findings in normal and T- and B-celldeficient mice. *Critical Care Medicine*.

[9] Hotchkiss RS, Swanson PE, Freeman BD (1999). Apoptotic cell death in patients with sepsis, shock, and multiple organ dysfunction. *Critical Care Medicine*.

[10] Coursin DB, Coursin DB, Maccioli GA (2001). Dexmedetomidine. *Current Opinion in Critical Care*.

[11] Ayoglu H, Gul S, Hanci V (2010). The effects of dexmedetomidine dosage on cerebral vasospasm in a rat subarachnoid haemorrhage model. *Journal of Clinical Neuroscience*.

[12] Taniguchi T, Kidani Y, Kanakura H, Takemoto Y, Yamamoto K (2004). Effects of dexmedetomidine on mortality rate and inflammatory responses to endotoxin-induced shock in rats. *Critical Care Medicine*.

[13] Sanders RD, Sun P, Patel S, Li M, Maze M, Ma D (2010). Dexmedetomidine provides cortical neuroprotection: impact on anaesthetic-induced neuroapoptosis in the rat developing brain. *Acta Anaesthesiologica Scandinavica*.

[14] Wells S, Trower C, Hough TA, Stewart M, Cheeseman MT (2009). Urethral obstruction by seminal coagulum is associated with medetomidine-ketamine anesthesia in male mice on C57BL/6J and mixed genetic backgrounds. *Journal of the American Association for Laboratory Animal Science*.

[15] Koca U, Olguner Ç, Karci A (2005). Antithrombin III pretreatment reduces neutrophil recruitment into the lung in a rat model of abdominal sepsis. *Acta Anaesthesiologica Scandinavica*.

[16] Hong YL, Yeh SL, Chang CY, Hu ML (2000). Total plasma malondialdehyde levels in 16 Taiwanese college students determined by various thiobarbituric acid tests and an improved high-performance liquid chromatography-based method. *Clinical Biochemistry*.

[17] Albaiceta GM, Gutiérrez-Fernández A, Parra D (2008). Lack of matrix metalloproteinase-9 worsens ventilator-induced lung injury. *American Journal of Physiology*.

[18] Yasuda H, Yuen PST, Hu X, Zhou H, Star RA (2006). Simvastatin improves sepsis-induced mortality and acute kidney injury via renal vascular effects. *Kidney International*.

[19] Tüzün F, Gencpınar P, Ozbal S (2012). Neuroprotective effect of neotrofin in a neonatal rat model of periventricular leukomalacia. *Neuroscience Letters*.

[20] Wichterman KA, Baue AE, Chaudry IH (1980). Sepsis and septic shock: a review of laboratory models and a proposal. *Journal of Surgical Research*.

[21] Seely KA, Holthoff JH, Burns ST (2011). Hemodynamic changes in the kidney in a pediatric rat model of sepsis-induced acute kidney injury. *American Journal of Physiology*.

[22] Wang Z, Holthoff JH, Seely KA, Pathak E, Spencer HJ, Mayeux PR (2012). Development of oxidative stress in the peritubular capillary microenvironment mediates sepsis-induced renal microcirculatory failure and acute kidney injury. *American Journal of Pathology*.

[23] Messaris E, Memos N, Chatzigianni E (2008). Apoptotic death of renal tubular cells in experimental sepsis. *Surgical Infections*.

[24] Wyllie AH (1980). Glucocorticoid-induced thymocyte apoptosis is associated with endogenous endonuclease activation. *Nature*.

[25] Aunapuu M, Kokk K, Tapfer H, Roosaar P, Talvic R, Liigant A (2004). Morphological and ultrastructural changes in the rat kidney after experimental sepsis. *Tsitologiia*.

[26] Gu J, Sun P, Zhao H (2011). Dexmedetomidine provides renoprotection against ischemia-reperfusion injury in mice. *Critical Care*.

[27] Kocoglu H, Ozturk H, Ozturk H, Yilmaz F, Gulcu N (2009). Effect of dexmedetomidine on ischemia-reperfusion injury in rat kidney: a histopathologic study. *Renal Failure*.

[28] Sanders RD, Xu J, Shu Y (2009). Dexmedetomidine attenuates isoflurane-induced neurocognitive impairment in neonatal rats. *Anesthesiology*.

[29] Park JH, Hwang IC, Ha N (2011). Effects of the anti-sepsis drug, (S)-1-(*α*-naphthylmethyl)-6,7- dihydroxy-1,2,3,4-tetrahydroisoquinoline (CKD-712), on mortality, inflammation, and organ injuries in rodent sepsis models. *Archives of Pharmacal Research*.

[30] Liu LX, Hu ZJ, Li Y, Su J, Huo Y, Fan LQ (2010). The effect of caspase-3 inhibitor on the concentrations of serum inflammatory cytokines in sepsis related acute kidney injury induced by peritoneal cavity infection in mice. *Chinese Critical Care Medicine*.

[31] Bellomo R, Kellum JA, Ronco C (2004). Defining acute renal failure: physiological principles. *Intensive Care Medicine*.

[32] Moran SM, Myers BD (1985). Course of acute renal failure studied by a model of creatinine kinetics. *Kidney International*.

[33] de Freitas DG, Picton M (2009). Clinical assessment of renal function. *Anaesthesia and Intensive Care Medicine*.

[34] Waikar SS, Betensky RA, Bonventre JV (2009). Creatinine as the gold standard for kidney injury biomarker studies. *Nephrology Dialysis Transplantation*.

[35] Wang W, Falk SA, Jittikanont S, Gengaro PE, Edelstein CL, Schrier RW (2002). Protective effect of renal denervation on normotensive endotoxemia-induced acute renal failure in mice. *American Journal of Physiology*.

[36] Schrier RW, Wang W (2004). Acute renal failure and sepsis. *New England Journal of Medicine*.

[37] Cumming AD, Kline R, Linton AL (1988). Association between renal and sympathetic responses to nonhypotensive systemic sepsis. *Critical Care Medicine*.

[38] Andén NE, Corrodi H, Fuxe K (1970). Evidence for a central noradrenaline receptor stimulation by clonidine. *Life Sciences*.

[39] Moura E, Afonso J, Hein L, Vieira-Coelho MA (2006). *α*
_2_-adrenoceptor subtypes involved in the regulation of catecholamine release from the adrenal medulla of mice. *British Journal of Pharmacology*.

[40] Taoda M, Adachi YU, Uchihashi Y, Watanabe K, Satoh T, Vizi ES (2001). Effect of dexmedetomidine on the release of [^3^H]-noradrenaline from rat kidney cortex slices: characterization of *α*
_2_-adrenoceptor. *Neurochemistry International*.

[41] Billings FT, Chen SWC, Kim M (2008). *α*
_2_-Adrenergic agonists protect against radiocontrast-induced nephropathy in mice. *American Journal of Physiology*.

[42] Devarajan P (2007). Emerging biomarkers of acute kidney injury. *Contributions to Nephrology*.

[43] Dent CL, Ma Q, Dastrala S (2007). Plasma neutrophil gelatinase-associated lipocalin predicts acute kidney injury, morbidity and mortality after pediatric cardiac surgery: a prospective uncontrolled cohort study. *Critical Care*.

[44] Bagshaw SM, Bennett M, Haase M (2010). Plasma and urine neutrophil gelatinase-associated lipocalin in septic versus non-septic acute kidney injury in critical illness. *Intensive Care Medicine*.

[45] Han M, Li Y, Liu M, Li Y, Cong B (2012). Renal neutrophil gelatinase associated lipocalin expression in lipopolysaccharide-induced acute kidney injury in the rat. *BMC Nephrology*.

[46] Paragas N, Qiu A, Zhang Q (2011). The Ngal reporter mouse detects the response of the kidney to injury in real time. *Nature Medicine*.

[47] Mårtensson J, Bell M, Oldner A, Xu S, Venge P, Martling CR (2010). Neutrophil gelatinase-associated lipocalin in adult septic patients with and without acute kidney injury. *Intensive Care Medicine*.

[48] Ozdulger A, Cinel I, Koksel O (2003). The protective effect of N-acetylcysteine on apoptotic lung injury in cecal ligation and puncture-induced sepsis model. *Shock*.

[49] Şener G, Toklu H, Ercan F, Erkanli G (2005). Protective effect of *β*-glucan against oxidative organ injury in a rat model of sepsis. *International Immunopharmacology*.

[50] Gül M, Ayan M, Seydanoğlu A (2011). The effect of N-acetyl cysteine on serum glutathione, TNF-*α* and tissue malondialdehyde levels in the treatment of sepsis. *Ulusal Travma ve Acil Cerrahi Dergisi*.

[51] Giamarellos-Bourboulis EJ, Grecka P, Dionyssiou-Asteriou A, Giamarellou H (1998). In vitro activity of polyunsaturated fatty acids on Pseudomonas aeruginosa: relationship to lipid peroxidation. *Prostaglandins Leukotrienes and Essential Fatty Acids*.

[52] Uysal HY, Cuzdan SS, Kayíran O (2012). Preventive effect of dexmedetomidine in ischemia-reperfusion injury. *Journal of Craniofacial Surgery*.

[53] Hanci V, Erol B, Bektaş S (2010). Effect of dexmedetomidine on testicular torsion/detorsion damage in rats. *Urologia Internationalis*.

[54] Ayoglu H, Gul S, Hanci V (2010). The effects of dexmedetomidine dosage on cerebral vasospasm in a rat subarachnoid haemorrhage model. *Journal of Clinical Neuroscience*.

[55] Eser O, Cosar M, Yaman M, Mollaoglu H, Songur A, Buyukbas S (2008). The influence of dexmedetomidine on ischemic rat hippocampus. *Brain Research*.

[56] Cinel I, Ark M, Dellinger P (2012). Involvement of Rho kinase (ROCK) in sepsis-induced acute lung injury. *Journal of Thoracic Disease*.

[57] Kono Y, Inomata M, Hagiwara S, Shiraishi N, Noguchi T (2012). A newly synthetic vitamin e derivative, E-Ant-S-GS, attenuates lung injury caused by cecal ligation and puncture-induced sepsis in rats. *Surgery*.

[58] Wu Y-X, Wu D-W, Peng M-M, Zhao L-K (2011). The effect of low-dose hydrocortisone on the expression of glucocorticoid receptor alpha of the septic kidney and its protective effect on kidney in rat. *Chinese Critical Care Medicine*.

[59] Yan G-T, Xue H, Lin J, Hao X-H, Zhang K, Wang L-H (2006). Leptin protects sepsis-induced renal injury and research for its mechanism. *Chinese Critical Care Medicine*.

[60] Chopra M, Reuben JS, Sharma AC (2009). Acute lung injury:apoptosis and signaling mechanisms. *Experimental Biology and Medicine*.

[61] Rinaldo JE, Henson JE, Dauber JH, Henson PM (1985). Role of alveolar macrophages in endotoxin-induced neutrophilic alveolitis in rats. *Tissue and Cell*.

[62] Goya T, Abe M, Shimura H, Torisu M (1992). Characteristics of alveolar macrophages in experimental septic lung. *Journal of Leukocyte Biology*.

[63] Charavaryamath C, Janardhan KS, Caldwell S, Singh B (2006). Pulmonary intravascular monocytes/macrophages in a rat model of sepsis. *Anatomical Record A*.

[64] Shi Q-Q, Wang H, Fang H (2012). Dose-response and mechanism of protective functions of selective alpha-2 agonist dexmedetomidine on acute lung injury in rats. *Saudi Medical Journal*.

[65] Szelényi J, Kiss JP (2000). Differential involvement of sympathetic nervous system and immune system in the modulation of TNF-*α* production by *α*
_2_- and *β*-adrenoceptors in mice. *Journal of Neuroimmunology*.

[66] Van Lanschot JJB, Mealy K, Jacobs DO, Evans DA, Wilmore DW (1991). Splenectomy attenuates the inappropriate diuresis associated with tumor necrosis factor administration. *Surgery Gynecology and Obstetrics*.

[67] Cunningham PN, Dyanov HM, Park P, Wang J, Newell KA, Quigg RJ (2002). Acute renal failure in endotoxemia is caused by TNF acting directly on TNF receptor-1 in kidney. *Journal of Immunology*.

[68] Holubec H, Payne CM, Bernstein H (2005). Assessment of apoptosis by immunohistochemical markers compared to cellular morphology in ex vivo-stressed colonic mucosa. *Journal of Histochemistry and Cytochemistry*.

[69] Gobe G (2009). Identification of apoptosis in kidney tissue sections. *Methods in Molecular Biology*.

[70] Cart NJ (2000). M30 expression demonstrates apoptotic cells, correlates with in situ end-labeling, and is associated with Ki-67 expression in large intestinal neoplasms. *Archives of Pathology and Laboratory Medicine*.

[71] van Grieken NCT, Meijer GA, Zur Hausen A, Meuwissen SGM, Baak JPA, Kuipers EJ (2003). Increased apoptosis in gastric mucosa adjacent to intestinal metaplasia. *Journal of Clinical Pathology*.

[72] Roth GA, Krenn C, Brunner M (2004). Elevated serum levels of epithelial cell apoptosis-specific cytokeratin 18 neoepitope M30 in critically ill patients. *Shock*.

[73] Leers MPG, Kölgen W, Björklund V (1999). Immunocytochemical detection and mapping of a cytokeratin 18 neo- epitope exposed during early apoptosis. *Journal of Pathology*.

[74] Perl M, Chung CS, Perl U (2007). Fas-induced pulmonary apoptosis and inflammation during indirect acute lung injury. *American Journal of Respiratory and Critical Care Medicine*.

[75] Wheeler DS (2009). Death to sepsis: targeting apoptosis pathways in sepsis. *Critical Care*.

[76] Oliver L, Vallette FM (2005). The role of caspases in cell death and differentiation. *Drug Resistance Updates*.

[77] Jo SK, Cha DR, Cho WY (2002). Inflammatory cytokines and lipopolysaccharide induce fas-mediated apoptosis in renal tubular cells. *Nephron*.

[78] Meßmer UK, Briner VA, Pfeilschifter J (1999). Tumor necrosis factor-*α* and lipopolysaccharide induce apoptotic cell death in bovine glomerular endothelial cells. *Kidney International*.

[79] Du C, Guan Q, Yin Z, Zhong R, Jevnikar AM (2005). IL-2-mediated apoptosis of kidney tubular epithelial cells is regulated by the caspase-8 inhibitor c-FLIP. *Kidney International*.

[80] Hengartner MO (2000). The biochemistry of apoptosis. *Nature*.

[81] Qiao H, Sanders RD, Ma D, Wu X, Maze M (2009). Sedation improves early outcome in severely septic Sprague Dawley rats. *Critical Care*.

[82] Engelhard K, Werner C, Eberspächer E (2003). The effect of the *α*
_2_-agonist dexmedetomidine and the N-methyl-D-aspartate antagonist S(+)-ketamine on the expression of apoptosis-regulating proteins after incomplete cerebral ischemia and reperfusion in rats. *Anesthesia and Analgesia*.

[83] Hotchkiss RS, Nicholson DW (2006). Apoptosis and caspases regulate death and inflammation in sepsis. *Nature Reviews Immunology*.

[84] Hotchkiss RS, Karl IE (2003). The pathophysiology and treatment of sepsis. *New England Journal of Medicine *.

[85] Dahmani S, Paris A, Jannier V (2008). Dexmedetomidine increases hippocampal phosphorylated extracellular signal-regulated protein kinase 1 and 2 content by an *α*
_2_-adrenoceptor- independent mechanism: evidence for the involvement of imidazoline I1 receptors. *Anesthesiology*.

